# Differential item functioning of the Geriatric Depression Scale‐short form in the NACC dataset

**DOI:** 10.1002/alz.71114

**Published:** 2026-02-11

**Authors:** Brenna N. Renn, Chad L. Cross, Ishrat Zaman, Katie T. Singsank, Kimberly Cobos, Samantha E. John

**Affiliations:** ^1^ Department of Psychology University of Nevada Las Vegas Nevada USA; ^2^ School of Veterinary Medicine Texas Tech University School of Veterinary Medicine Amarillo Texas USA; ^3^ Department of Epidemiology & Biostatistics School of Public Health, University of Nevada Las Vegas Nevada USA; ^4^ Department of Brain Health Kirk Kerkorian School of Medicine, University of Nevada Las Vegas Nevada USA

**Keywords:** Alzheimer's disease and related dementias, cognitive impairment, Differential item functioning, multiple indicator multiple cause modeling

## Abstract

**INTRODUCTION:**

This study examined differential item functioning of the Geriatric Depression Scale – Short Form (GDS‐SF) in the National Alzheimer's Coordinating Center (NACC) Uniform Data Set (UDS) to identify potential variables that produce measurement bias.

**METHODS:**

Data from 14077 individuals’ first NACC visit were analyzed. Multiple indicator, multiple causes (MIMIC) models assessed differential item functioning (DIF) of the 15‐item GDS‐SF across race, Hispanic ethnicity, primary language, sex, and cognitive status (Clinical Dementia Rating [CDR] scale scores), while adjusting for educational attainment.

**RESULTS:**

Participants were on average 73 (SD = 9.1) years old and 54.4% women. The majority (13 of 15) of the GDS‐SF items demonstrated DIF. For many items, participants with any level of CDR cognitive impairment were more likely to endorse depressive symptoms.

**DISCUSSION:**

Findings indicate the presence of widespread DIF by cognitive impairment severity such that individuals with even mild cognitive impairment may respond differently to certain items on this measure.

**Highlights:**

- The Geriatric Depression Scale – Short Form (GDS‐SF) showed differential item functioning (DIF) in 13 of 15 items across demographic and cognitive groups.
- Only two items—hopelessness and worthlessness—were invariant across all groups.
- Cognitive status (Clinical Dementia Rating [CDR]) most strongly influenced item endorsement patterns.
- Our study used a large, diverse sample (National Alzheimer's Coordinating Center Uniform Data Set [NACC UDS]) and robust DIF analytic methods.
- Findings highlight both reliable and problematic GDS‐SF items for older adults.

## 1 BACKGROUND

Depression undermines healthy aging by impairing self‐care and daily functioning, reducing well‐being, and increasing risk of progression to and acceleration of Alzheimer's disease (AD).^1^, ^2^ Depression is an important modifiable risk factor for dementia^3^ and is common in individuals with neurodegenerative disorders, with prevalence estimates ranging from 38% in AD to 50% among vascular dementia cases.^4^ In contrast, national estimates from the general community‐dwelling US population aged 65 and older show a much lower prevalence of major depressive disorder, at approximately 3.0%.^5^

Depression prevalence also varies across demographic characteristics. Women are more likely than men to report and be diagnosed with depression across the lifespan.^5^ Some non‐representative studies suggest that African American older adults may have lower prevalence of depression than their non‐Hispanic White counterparts.^6^ In contrast, data from Centers for Medicare and Medicaid Services indicate that Hispanic/Latino and Black/African American adults aged 65 and older have higher odds of screening positively for depression compared to their non‐Hispanic White counterparts.^7^ Persistent health disparities underscore the need for depression screening tools that are unbiased across demographic groups.

Variations in prevalence may reflect true differences in depression epidemiology but could also arise from measurement bias, such as differential item functioning (DIF). DIF occurs when individuals from different groups (e.g., sex, race, ethnicity) have different probabilities of endorsing specific items on a measure, despite equivalent underlying levels of the construct.^8^ After accounting for an individual's depression severity, other characteristics should not influence the likelihood of item endorsement.

The Geriatric Depression Scale (GDS)^9^ is a self‐report tool developed to screen depression among adults aged 55 and older; the original form and 15‐item short form^10^ are commonly used in cognitive aging research. Despite their widespread use, their validity in racially and ethnically marginalized populations and individuals with cognitive impairment has been questioned and evaluated. Umucu and colleagues^11^ explored the ability of the GDS to detect symptoms among ethnoracial groups in an Alzheimer's Disease Research Center (ADRC) cohort and found that African American participants reported more symptoms than non‐Hispanic White participants. Fieo and colleagues examined DIF of the GDS – Short Form (GDS‐SF) across four longitudinal aging studies, including select ADRCs, and found DIF by cognitive status, though the effect on total scores was negligible.^12^ Midden and Mast found partial measurement invariance of the GDS‐SF with DIF in two items according to presence or absence of cognitive impairment, though their sample was small, limiting power and increasing the likelihood of unstable parameter estimates.^13^, ^14^ Chiesi and colleagues identified DIF for two GDS‐SF items (memory problems and fear of something bad happening) in an Italian sample. Although their sample included those with cognitive impairment, more work is needed across cognitively intact and mildly impaired individuals to aid in replication and interpretability.^15^ If measurement properties are not examined within diverse patients, then assessment tools may contribute to misdiagnosis or misrepresent the efficacy of a given treatment approach in trials.

To best identify and equivalently measure depression across groups,  scores must be comparable across individuals of varying characteristics.^16^ Cultural perceptions of disease, severity of impairment, and patient values may contribute to different interpretation of symptoms and item response.^17^, ^18^ Assessing DIF among large, heterogeneous research samples is necessary to ensure generalizability of assessment and improve patient‐centered care. The COnsensus‐based Standards for the selection of health Measurement INstruments (COSMIN) provides guidelines for evaluating and selecting patient‐reported outcome measures, and recommends establishing cross‐cultural validity through measurement invariance testing and DIF approaches.^19^ To this aim, we applied multiple indicator multiple cause (MIMIC) structural equation modeling to test for DIF across GDS‐SF items by cognitive status, race, ethnicity, sex, and primary language, while adjusting for education, in the National Alzheimer's Coordinating Center (NACC) cohort. NACC aggregates data across the nationwide network of ADRCs to create one of the largest U.S. datasets related to cognitive aging. The size and diversity of the sample provide a unique opportunity to extend prior DIF research with greater emphasis on cognitive ability and consideration of additional demographic characteristics.

## 2 METHODS

### 2.1 Setting and data characteristics

This study is a retrospective cross‐sectional analysis of the publicly available NACC Uniform Data Set (UDS). The NACC database includes data collected at participating National Institute on Aging (NIA) ‐funded ADRCs across the US. Participants are self‐referred, family‐referred, physician‐referred, or recruited from the community by each ADRC. The UDS was implemented in September 2005 to prospectively gather standardized clinical data from study participants. NACC‐UDS data collection procedures have been described in detail elsewhere.^20^, ^21^, ^22^ In brief, participants complete an annual evaluation including clinical exam, neuropsychological testing, and completion of clinician‐administered and self‐reported measures. Participants are consented under institution‐specific institutional review board (IRB) ‐approved protocols for data collection procedures at their respective ADRC and for inclusion into the NACC data repository. The University of Washington IRB is the primary IRB for research using the deidentified NACC database. Data for the present study were obtained through request and data use agreement with the NACC UDS website (https://naccdata.org/) and taken from the data freeze of June 2023. To facilitate study replication and increase methodological transparency, we include exact NACC UDS variable names within the sections below.

### 2.2 Participant and sample selection

The present study only uses data from the participants’ first visit and includes participants from all versions of the NACC UDS, v1 – v3.2. From the original data file (*N* = 44,714), we selected a sample for analysis that retained only those participants aged 55+ (NACCAGE), who were assigned a diagnosis (NACCUDSD) of cognitively normal, mild cognitive impairment, or dementia, and Clinical Dementia Rating (CDR) Global scores of 0–1 (i.e., those classified as “no impairment”, “questionable impairment” or “mild impairment” only on CDRGLOB). Individuals with CDR scores of 2 (moderate impairment) or 3 (severe impairment) were not included in the MIMIC analyses because the validity of the GDS in cognitively impaired older adult subjects has been questioned and due to general concerns of reliability and validity related to self‐reported depression assessment in addition to potential DIF. Owing to the large size of the database, we elected to utilize data only from participants who completed GDS‐SF responses across all 15 questions; hence, all identified participants with incomplete data on any of the items of the GDS‐SF were excluded from the sample to remove response bias as a possible confounding factor in our analyses. No missing data replacement was used. The final dataset including participants with completed GDS‐SF was *n* = 14,077. Of these, 6.4% were missing information relative to one of the modeled covariates, years of education (see below); hence, models were assessed with the complete data from 13,177 participants.

**RESEARCH IN CONTEXT**

1. **Systematic review**: The authors reviewed the literature (e.g., PubMed) to identify prior studies evaluating the psychometric properties of the Geriatric Depression Scale – Short Form (GDS‐SF), with a focus on differential item functioning (DIF). Most prior studies used limited samples and tested only a narrow range of demographic variables, restricting generalizability.
2. **Interpretation**: Our findings expand on previous work by using a large and diverse sample from the National Alzheimer's Coordinating Center (NACC) to test for DIF across race, ethnicity, sex, primary language, and cognitive status. Thirteen of 15 GDS‐SF items demonstrated DIF, with cognitive status exerting the greatest influence. Only two items were invariant across all covariates.
3. **Future directions**: Future studies should validate GDS‐SF DIF findings in clinically validated samples of those with and without depression. Investigations should also explore development of revised or adaptive GDS‐SF items for use across diverse cognitive and demographic groups.

### 2.3 Measures

#### 2.3.1 Demographic characteristics

Categorical demographic characteristics included race (White, Black or African American, American Indian or Alaska Native, Native Hawaiian or other Pacific Islander, Asian, Other (specify), unknown), ethnicity (Hispanic/Latino or non‐Hispanic/Latino), sex (binary male/female; gender not available as it was not added until later datasets), and primary language (English, Spanish, Mandarin, Cantonese, Russian, Japanese, Other primary language (specify), unknown). We additionally included information on educational attainment as a surrogate measure of socio‐economic status as detailed in the analysis section below. Further, to enhance our description of the participants utilized in the analysis, we summarized data associated with living arrangements and marital status.

#### 2.3.2 Cognitive measures

To determine cognitive status and sample selection, we utilized the CDR^©^ Dementia Staging Instrument Global score. The CDR measures six domains (memory, orientation, judgement and problem solving, community affairs, home and hobbies, and personal care) of cognitive and functional performance related to AD and related dementias (ADRD). The CDR is used for the clinical diagnosis and characterization of ADRD. Possible scores include 0.0 no impairment, 0.5 questionable impairment, 1.0 mild impairment, 2.0 moderate impairment, to 3.0 severe impairment.^23^ Given concerns with the validity of self‐report instruments generally and the GDS specifically in individuals with moderate or severe dementia,^24^, ^25^ we retained only those participants with CDR Global scores of 0, 0.5, and 1. This decision affords a spectrum of cognitive ability in which to test DIF, from individuals with normal cognition through those with mild cognitive impairment and ADRD, while minimizing the nonsystematic errors in responding such as poor insight, poor recall, or variable responding.

For descriptive purposes, we also included total scores from the Mini‐Mental State Examination (MMSE)^26^ and Montreal Cognitive Assessment (MoCA).^27^ Both are widely used, well‐validated screening measures for cognitive impairment. The MMSE includes items assessing orientation to time and place, immediate and delayed recall, attention and calculation, language, and visual construction ability. The MoCA includes items assessing the above domains as well as additional measures of frontal executive function. Both scales are scored from 0 to 30, with higher scores indicating better cognitive performance.

#### 2.3.3 Depression measure

The primary depression measure in the NACC UDS is the 15‐item GDS‐SF.^10^ This self‐report depression screening tool was derived from the original 30‐item version to reduce respondent burden, particularly in individuals experiencing fatigue or concentration difficulties. The GDS‐SF includes 15 self‐reported items suggestive of depression among older adults. Respondents indicate whether they have experienced each symptom during the past week on a dichotomous yes (1) or no (0) response scale. Possible summary scores range from 0 to 15 (after select items are reverse‐coded). A threshold of ≥ 5 recommends further investigation into the presence of a depressive episode.^10^ In the NACC dataset, the GDS is administered if, according to the clinician's best judgment, the participant can complete the GDS. Because the characteristics of depression captured by this scale are largely subjective (e.g., feeling of satisfaction with one's life), there is no informant or collateral report used in NACC.^28^, ^29^

### 2.4 Statistical analysis

**FIGURE 1:**
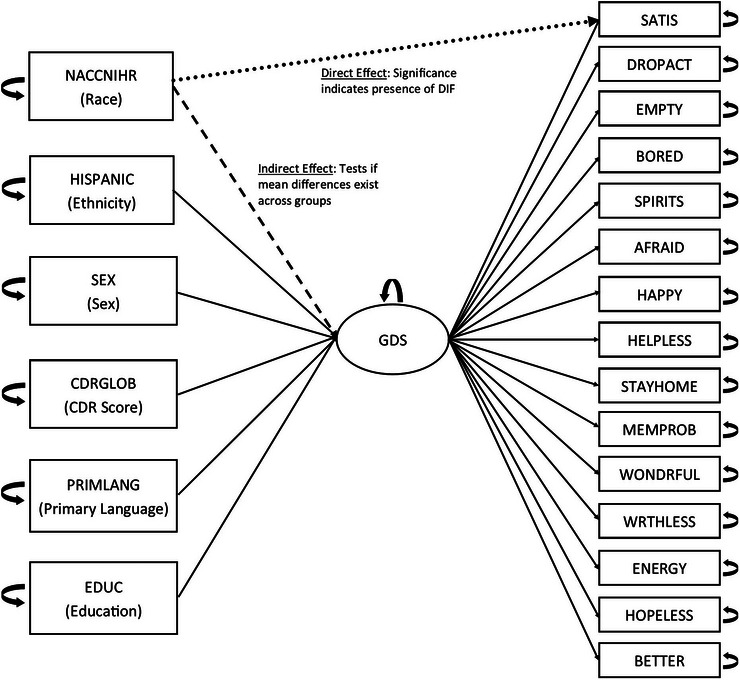
Multiple indicator multiple cause (MIMIC) models used to probe influence of demographic and cognitive characteristics. *Note*: In this modeling context, the depression construct is modeled using the 15 items of the Geriatric Depression Scale – Short Form (GDS‐SF). In turn, each potential covariate evaluated for differential item functioning (DIF) was entered into the model to create direct and indirect effects. A significant direct effect in this case suggests that there is a potential difference among the groups represented by the covariates on the model latent trait, and hence the potential presence of DIF. Per structural equation modeling (SEM) conventions, rectangles represent observed (i.e., directly measured) variables and the oval represents the latent variable of depression as conceptualized by the GDS. Observed variable names appear exactly as listed in the National Alzheimer's Coordinating Center (NACC) Uniform Data Set (UDS). The observed variables along the lefthand side are the multiple covariates used to interrogate DIF in the 15 GDS‐SF items (presented along the righthand side). GDS‐SF items appear in the same order as on the published screening tool. Abbreviations. SATIS, Are you basically satisfied with your life?; DROPACT, Have you dropped many of your activities and interests?; EMPTY, Do you feel that your life is empty?; BORED, Do you often get bored?; SPIRITS, Are you in good spirits most of the time?; AFRAID, Are you afraid that something bad is going to happen to you?; HAPPY, Do you feel happy most of the time?; HELPLESS, Do you often feel helpless?; STAYHOME, Do you prefer to stay at home, rather than going out and doing new things?; MEMPROB, Do you feel you have more problems with memory than most people?; WONDRFUL, Do you think it is wonderful to be alive?; WRTHLESS, Do you feel pretty worthless the way you are now?; ENERGY, Do you feel full of energy?; HOPELESS, Do you feel that your situation is hopeless?; BETTER, Do you think that most people are better off than you are?

We calculated descriptive statistics for all model variables as well as seven characterization variables; these were total MoCA score (MOCATOTS), total MMSE score (NACCMMSE), current antidepressant use (NACCADEP), living situation (NACCLIVS), level of independence (INDEPEND), type of residence (RESIDENC), and marital status (MARISTAT). Measurement invariance of a given scale can be evaluated using several approaches. Multi‐group confirmatory factor analysis (CFA) is often employed to test configural, metric, and scalar invariance across groups. Such assessment examines invariance at the scale level (across all items simultaneously). Differential item functioning (DIF) methods focus on the item level and thus pinpoint which items contribute to noninvariance. One strategy to assess DIF is the multiple indicators multiple causes (MIMIC) model, which incorporates group variables as covariates to detect noninvariance and its possible sources. In this study, we applied a MIMIC framework to explore potential DIF of the 15 GDS‐SF items. We followed the methodology outlined by Cheng, Shao, and Lathrop^30^ and Proitsi and colleagues^31^ for developing MIMIC models using structural equation models (SEM). In this modeling context, a potential covariate is added to the model, and its direct effect path to each item is estimated. A significant direct effect path indicates DIF (Figure 1). Our MIMIC analyses explored the influence of multiple covariates, including (with reference category underlined): (1) race (NACCNIHR), categorized as White, Black, Other race (inclusive of Asian/Asian American, Pacific Islander, and American Indian), and multiracial; (2) ethnicity (HISPANIC), categorized as Hispanic or not Hispanic; (3) sex (SEX), categorized as female or male; (4) global impairment rating on the Clinical Dementia Rating (CDRGLOB), categorized as no impairment (CDR = 0), questionable impairment (CDR = 0.5), or mild impairment (CDR = 1.0); and (5) primary language (PRIMLANG variable), categorized as English, Spanish, or Other. Variable categories, when exceeding two levels, were condensed relative to available sample sizes. Reference groups for each variable were identified as representative of those typically included in ADRD and measure development research and most representative of the original GDS validation samples.^9^, ^32^, ^33^ Additionally, we utilized years of educational attainment in the model as a surrogate measure of socio‐economic level. We utilized the <lavaan>^34^ and <semPlot>^35^ packages in R (v. 4.4.2) for SEM analyses as suggested by Chang and colleagues.^36^ We evaluated model fit using the comparative fit index (CFI), Tucker‐Lewis index (TLI), root mean square error of approximation (RMSEA), and the standardized root mean square residual (SRMR). Owing to the large number of models, we protected family‐wise error rate to no larger than α = 0.05 using Bonferroni‐adjustment of *p*‐values (α = 0.001 for individual tests). If DIF was indicated because of significance of the direct effect, we conducted follow‐up analyses of the individual significant items using binary logistic regression (i.e., the dichotomous response of 0 = item not endorsed versus 1 = item endorsed) on the dependent variable using the five categorical covariates and the continuous variable, years of education, to adjust the model. Betas in these models were exponentiated to calculate adjusted odds ratios (ORs) using the reference categories underlined above. A 95% confidence interval (CI) for the adjusted OR containing “1” indicates no significant difference was found between a given group and its reference category; conversely, 95% CIs not containing “1” are considered significant. It should be noted that 95% CIs are sensitive to standard error estimates so that it is possible that the MIMIC model suggested DIF, but that the binary logistic model was not able to capture this significance owing to estimation precision or because other important differences were not captured as they were related to an unknown or non‐modeled variable. These exploratory analyses are presented as potential directions for future hypothesis generation. Even though the purpose of the SEM models was for identifying potential DIF across covariates and not for generating modeled estimates or predictive modeling, per se, we conducted a sensitivity analysis of the SEM to examine the likelihood of a potential unmeasured covariate and the stability of the SEM path results. For this sensitivity analysis, we included a Gaussian‐parameterized “phantom” random variable^37^ into the SEM model through a range of possible effect sizes (e.g., 0.1, 0.2, etc.). From each of these generated models, we examined the path estimate magnitude and statistical significance for each modeled variable as well as the Akaike Information Criteria (AIC) and the same model fit statistics as described above.

## 3 RESULTS

### 3.1 Demographic and sample summaries

Table 1 depicts characteristics of the final analytic sample relative to the MIMIC model variables. Supporting tables include additional descriptive summaries to help contextualize the sample, including cognitive and depression characteristics (Table S1), living situation (Table S2), level of independence (Table S3), type of residence (Table S4), and marital status (Table S5). The mean age of participants was 73 years (SD = 9.1 years; 95% CI: 72.9, 73.1). Ages ranged from 55 to 109 years. Age data were neither skewed (*γ* = 0.17, SE = 0.018) nor kurtotic (*κ* = ‐0.41, SE = 0.035). The mean educational attainment was 15 years (SD = 2.4 years; 95% CI: 14.9, 15.1). Years of education was not skewed (*γ* = ‐0.70, SE = 0.021), but displayed a slight positive kurtosis (*κ* = 1.28, SE = 0.043) with most participants ranging from 12‐20 years of education and few in the tails; however, observation of the Q‐Q plot did not suggest issues with normality of the variable. Participants were 54.4% (*n* = 7661) female and 45.6% (*n* = 6416) male. Item endorsements varied among each of the covariates used in the models, suggesting that DIF may be a useful evaluation of this instrument in this population (Tables S6–S10). Average GDS‐SF scores were 2.07 (SD = 2.53, Mdn = 1, IQR 3, total score range 0–15), and 13.8% (*n* = 1942) of the sample scored within the clinical range (GDS‐SF ≥ 5). Descriptive statistics for MoCA and MMSE indicated the lowest scores among those with mild impairment that trended upward within less impaired participants. Antidepressant use varied among groups, with highest use reported among White individuals and those with mild impairment on the CDR. The lowest antidepressant use was reported among Black/African American individuals and those captured in the Other race category.

**TABLE 1:** Modeled sample characteristics.

Variable	Category	N (%)
Race	White	10652 (79.9)
	Black or African American	1867 (13.9)
	Multiracial	360 (2.9)
	Other	444 (3.3)
Ethnicity	Not Hispanic	12913 (92.1)
	Hispanic	1106 (76.9)
Sex	Male	6416 (45.6)
	Female	7661 (54.4)
CDR	No Impairment	6053 (43.0)
	Questionable impairment	5432 (38.6)
	Mild impairment	2592 (18.4)
Primary language	English	12916 (91.8)
	Spanish	766 (5.4)
	Other	393 (2.8)

*Note*: Descriptive characteristics of the categorical modeled variables from the MIMIC models, showing the condensed categories within each variable.

Abbreviations: CDR, Clinical Dementia Rating Dementia Staging Instrument Global score.

### 3.2 MIMIC models

The MIMIC model is presented in Figure 1. All SEM models were significant compared to baseline intercept‐only models (all *p* < 0.001). Model fit statistics were acceptable. Fit indices were all quite good, with CFI = 0.986, TLI = 0.984, RMSEA = 0.053, and SRMR = 0.038 indicating good fit and confirming that the GDS‐SF items measure a unitary and interpretable construct. DIF was indicated disproportionately across GDS items (Table 2). For example, items assessing hopelessness (“Do you feel that your situation is hopeless?”) and worthlessness (“Do you feel worthless the way you are now?”) did not have any indication of influence from the examined covariates, whereas the item assessing energy (“Do you feel full of energy?”) indicated DIF across all variables modeled. Primary language and race did not consistently demonstrate DIF (only 3 and 4 items, respectively). More items demonstrated DIF when modeled with sex (6 items), ethnicity (8 items), and in particular, CDR global score (12 of 15 items significant).

**TABLE 2:** MIMIC model results examining DIF for demographic and cognitive characteristics.

GDS‐SF Items	NACCNIHR (Race)		HISPANIC (Ethnicity)		SEX (Sex)		CDRGLOB (CDR Score)		PRIMLANG (Primary Language)	
	Z	*p*‐value	Z	*p*‐value	Z	*p*‐value	Z	*p*‐value	Z	*p*‐value
SATIS	−**4.095**	**<0.001**	−**3.306**	**0.001**	1.319	0.187	−**6.154**	**<0.001**	−**4.408**	**<0.001**
DROPACT	1.902	0.057	**5.168**	**<0.001**	−1.896	0.058	**13.514**	**<0.001**	−0.683	0.494
EMPTY	0.888	0.374	**6.448**	**<0.001**	**5.184**	**<0.001**	−**2.969**	**0.003**	1.185	0.236
BORED	−0.850	0.395	2.174	0.030	−**4.406**	**<0.001**	**8.967**	**<0.001**	−1.721	0.085
SPIRITS	−0.923	0.356	0.702	0.483	−0.383	0.701	−**8.836**	**<0.001**	0.997	0.319
AFRAID	**4.626**	**<0.001**	**3.413**	**0.001**	1.771	0.077	−**4.630**	**<0.001**	**6.034**	**<0.001**
HAPPY	−1.872	0.061	−1.258	0.208	2.474	0.013	−**12.965**	**<0.001**	1.277	0.202
HELPLESS	1.180	0.238	−0.017	0.986	2.477	0.013	**4.053**	**<0.001**	0.362	0.717
STAYHOME	1.484	0.138	**0.254**	**0.001**	−**5.278**	**<0.001**	0.540	0.589	−0.332	0.740
MEMPROB	0.472	0.637	−**5.553**	**<0.001**	−**9.612**	**<0.001**	**34.403**	**<0.001**	−0.848	0.397
WONDRFUL	0.118	0.906	−**6.395**	**<0.001**	2.835	0.005	−**8.508**	**<0.001**	0.381	0.703
WRTHLESS	0.257	0.797	−0.620	0.536	0.062	0.951	2.177	0.029	−0.231	0.817
ENERGY	−**3.696**	**<0.001**	−**6.935**	**<0.001**	**3.539**	**<0.001**	−**5.738**	**<0.001**	−**3.967**	**<0.001**
HOPELESS	−0.025	0.980	0.283	0.777	−2.095	0.036	−0.979	0.327	0.078	0.938
BETTER	**3.522**	**<0.001**	−0.228	0.819	−**4.063**	**<0.001**	**6.219**	**<0.001**	2.279	0.023

*Note*: Path z‐scores (“Z”) and *p*‐values are shown. Bold type highlights significant DIF paths based on Bonferroni‐correction. Demographic and cognitive characteristics are labeled with variable names from the NACC UDS dataset and descriptive labels. NACCNIHR, NACC derived variable for racial identity. HISPANIC, NACC variable for Hispanic ethnicity (yes/no).

Abbreviations: CDRGLOB, Clinical Dementia Rating Dementia Staging Instrument Global score; GDS‐SF, Geriatric Depression Scale – Short form; MIMIC, multiple indicator multiple cause; NACC, National Alzheimer's Coordinating Center; PRIMLANG, participant primary language.

Sensitivity analysis using a phantom variable to assess for the influence of potential missing confounders in the model provided interesting results. Models with increasing effect sizes had increasing AICs suggesting that the baseline model without the phantom variable was the most parsimonious solution. Additionally, CFI and TLI measures were within range (> 0.90) across all sensitivity models; however, RMSEA and SRMR increased as the phantom effect size increased, and some modeled paths that were significant in the baseline model became insignificant with increasing phantom effect size. In particular, sex and ethnicity were no longer significant in models with phantom effect sizes that are traditionally considered large (i.e., > 0.80). In summary, this suggests that the SEM model utilized in this study to investigate potential DIF seems stable with a small‐to‐moderate phantom covariate introduced into the model, but if an unmeasured covariate with a large effect size were introduced, the model paths may change to some degree. It is relatively unlikely that large‐effect confounders may be found among the measured variables of the NACC database we used in this study and which we did not account for in our model relative to DIF, though this remains a hypothesis‐generating result.

### 3.3 Post hoc models

**FIGURE 2:**
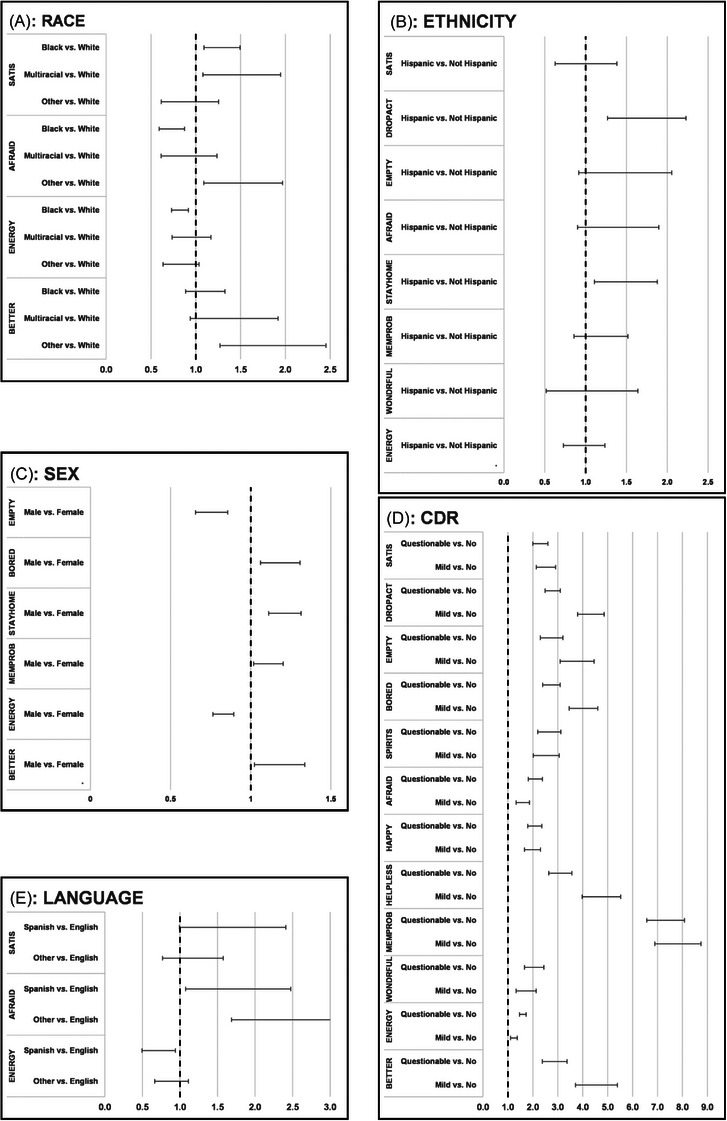
Post hoc forest plots demonstrating endorsement patterns. *Note*: Forest plots showing 95% confidence intervals for adjusted odds ratios (ORs) from logistic regression models for covariate categories demonstrating potential differential item functioning (DIF) in multiple indicator multiple cause (MIMIC) models. The vertical dotted line indicates equal odds of endorsing the response “1” between a given category and its reference group; significance is indicated when the 95% confidence interval (CI) does not overlap this vertical line. In panel 2A, results of the logistic regression models for covariate Race are considered, where White is the reference group. In panel 2B, results of the logistic regression models for covariate Ethnicity, where Not Hispanic is the reference group. In panel 2C, results of the logistic regression models for covariate Sex, where Female is the reference group. In panel 2D, results of the logistic regression models for covariate Clinical Dementia Rating (CDR), where No Impairment is the reference group. In panel 2E, results of the logistic regression models for covariate Primary Language, where English is the reference group. The 15 items of the Geriatric Depression Scale (GDS‐SF) appear exactly as listed in the National Alzheimer's Coordinating Center (NACC) Uniform Data Set (UDS). SATIS: Are you basically satisfied with your life?; DROPACT: Have you dropped many of your activities and interests?; EMPTY: Do you feel that your life is empty?; BORED: Do you often get bored?; SPIRITS: Are you in good spirits most of the time?; AFRAID: Are you afraid that something bad is going to happen to you?; HAPPY: Do you feel happy most of the time?; HELPLESS: Do you often feel helpless?; STAYHOME: Do you prefer to stay at home, rather than going out and doing new things?; MEMPROB: Do you feel you have more problems with memory than most people?; WONDRFUL: Do you think it is wonderful to be alive?; WRTHLESS: Do you feel pretty worthless the way you are now?; ENERGY: Do you feel full of energy?; HOPELESS: Do you feel that your situation is hopeless?; BETTER: Do you think that most people are better off than you are?

Post hoc logistic models were used to assess endorsement differences within each demographic variable. Forest plots provide visual details of adjusted odds ratio comparisons (Figure 2), where significant items may differ from those identified in Table 2, relative to the post‐hoc models’ ability to detect differences. First, in examining differences between racial groups, Black/African American and Multiracial individuals had higher odds than White respondents for endorsing the satisfaction item (*“*Are you basically satisfied with your life?”). Black/African American respondents were less likely, and the Other racial group more likely, than the White reference group to endorse “Are you afraid that something bad is going to happen to you?”. Black/African American respondents had lower odds relative to White respondents of responding to the energy item. Members of the Other racial group were more likely than White individuals to endorse the item, “Do you think that most people are better off than you are?”. Second, in examining differences by ethnicity, Hispanic individuals had higher odds relative to the non‐Hispanic group of responding affirmatively to two items: “Have you dropped many of your activities and interests?” and “Do you prefer to stay at home, rather than going out and doing things?”. Third, in evaluating the influence of sex, men had lower odds of endorsing the energy item as well as the item “Do you feel that your life is empty?”. Men had higher odds of endorsing items assessing boredom (“Do you often get bored?”), preference to stay at home (“Do you prefer to stay at home, rather than going out and doing things?”), subjective memory concerns (“Do you feel that you have more problems with memory than most?”), and the feeling that others are better off than they are. Fourth, examination of the 12 items with post hoc evidence of DIF among CDR groups found a nearly universal pattern, with individuals in both the questionable (CDR = 0.5) and mild impairment (CDR = 1.0) categories demonstrating higher odds of endorsing all items relative to those with no impairment (CDR = 0). Finally, examining differences by primary language revealed that Spanish speakers had higher odds than English speakers of endorsing a sense of satisfaction with their life, and lower odds of endorsing the energy item. Both Spanish and Other primary language speakers had higher odds compared to the English‐speaking reference group of feeling afraid something bad will happen.

## 4 DISCUSSION

We examined measurement invariance of the GDS‐SF within a large, diverse sample of older adult participants across relevant demographic characteristics that may bias measurement, including race, ethnicity, sex, primary language, and cognitive status, while adjusting for education. In our sample of NACC participants, 13 of 15 GDS‐SF items demonstrated DIF, depending on various respondent characteristics. Two items—one assessing hopelessness and one worthlessness—demonstrated invariance across all measured characteristics. Cognitive status, as measured by the CDR, exerted the greatest influence on item endorsement, with noninvariance among CDR groups for 12 items.

### 4.1 Item performance and demographic influence

Certain items evidenced noninvariance across multiple demographic characteristics. The item assessing energy (“Do you feel full of energy?”) indicated DIF across all demographic variables modeled. The items assessing satisfaction (“Are you basically satisfied with your life?”) and fear (“Are you afraid that something bad is going to happen to you?”) were susceptible to noninvariance across all covariates, except sex. Aside from these three items, primary language did not influence DIF in our sample. Race did not demonstrate a consistent influence, exerting DIF on the three items above as well as the item, “Do you think that most people are better off than you are?”. Post hoc testing did not reveal a consistent pattern of DIF by racial group across items. Modeling the influence of sex, ethnicity, and cognitive status within our MIMIC models resulted in 6, 8, and 12 items with DIF, respectively. Post hoc tests within sex demonstrated that differential item endorsement was item specific. In contrast, Hispanic ethnicity was associated with increased likelihood of item endorsement; however, only two of eight items remained significant in post hoc analysis. Post hoc testing of items influenced by the CDR revealed the most prominent and consistent effects in which questionable and mild cognitive impairment resulted in increased likelihood of item endorsement for all 12 of the model identified items. Although we did not model age as a covariate in our analysis, prior examinations of the GDS have confirmed the absence of DIF as a function of age.^15^, ^32^, ^38^

### 4.2 Subjective memory complaints and depression

Given the influence of cognitive status on most of the items of the GDS‐SF, it is perhaps not surprising that the greatest difference in endorsement among CDR groups occurred for the item assessing subjective memory (“Do you feel you have more memory problems than most?”). Prior papers have identified the memory item as problematic across different samples. Midden and Mast identified DIF for both the memory and energy items (“Do you feel full of energy?”) among older adults with and without cognitive impairment, despite finding general support of measurement invariance of the GDS‐SF.^14^ The memory item was particularly problematic within their sample, such that individuals with cognitive impairment had a lower probability of endorsment than individuals without impairment, independent of depression level. Dropping this item resulted in a greater proportion of older adults with cognitive impairment meeting the threshold for depression screening (GDS‐SF ≥ 5). Notably, their sample was composed primarily of non‐Hispanic White adults, and they defined cognitive status (cognitively impaired vs. unimpaired) at the 10th percentile of performance on the Mattis Dementia Rating Scale‐2.

Other investigations have also revealed DIF specific to the memory item. Most recently, Chiesi et al. identified DIF for two GDS‐SF items (memory problems and fear of something bad happening) based upon cognitive functioning among 1305 older adults in a longitudinal multicenter clinical‐based study in Italy.^15^ Marc and colleagues examined the GDS‐SF in 539 older adults receiving home care for medical conditions in New York.^32^ The authors observed DIF of the memory item by sex (men were more likely than women to endorse this item, controlling for total GDS score) and race and ethnicity (African American/Black and Hispanic respondents were more likely than White respondents to endorse this item, controlling for GDS score). Our analyses found DIF for the memory complaints item among ethnic groups (Hispanic/non‐Hispanic), sex, and most notably, cognitive status, though not always in the same direction as previous investigations, reinforcing the influence of sample, setting, and characteristics probed.

### 4.3 Clinical implications of depression screening with the GDS‐SF

Our and others’ findings suggest caution and additional interpretation when using the GDS‐SF in cognitively impaired samples, even among those with questionable or mild impairment. Diagnosis of depression among older adults, particularly those with cognitive impairment, is complicated by overlapping social, somatic, and cognitive symptoms.^39^, ^40^ The GDS‐SF avoids items based on somatic symptoms that may present in medical conditions common to aging, but assessing subjective memory complaints in older adults with cognitive impairment seems problematic and likely to render item endorsement unrelated to depression.^15^ Subjective memory complaints in the absence of any clinical evidence of cognitive impairment should be further explored as possibly related to depression. However, this assessment is complicated by the mounting evidence of a bidirectional relationship between depression and cognitive impairment^41^ and rendered all the more important by findings that mid‐ and later‐life depression can be a risk factor^42^, ^43^ and prodrome of ADRD.^44^

It is important that the GDS is used as intended–that is, as a screening instrument to identify individuals who may have depression; it does not provide a definite diagnosis. A positive screen on the GDS‐SF (total score ≥ 5), particularly in an older adult with cognitive impairment, should be further interrogated by a clinician to confirm or rule out a diagnosis and plan treatment accordingly.^9^ Even among cognitively normal older adults, clinicians should consider the influence of certain identity characteristics and further probe individuals to best understand their answers to specific items. Item endorsement is based on both symptom and item interpretation and may be indicative of underreporting of depression or presence of other related conditions. Given that the NACC relies on the GDS‐SF as a key measure of depression, other patient‐reported outcomes may enhance the validity of depression findings. Caregiver or informant report of neuropsychiatric symptoms, as through the Neuropsychiatric Inventory‐Questionnaire (NPI‐Q),^45^ could provide collateral report of the presence of depression in the UDS.^20^, ^22^, ^29^ Taken one step further, the Alzheimer's Disease Neuroimaging Initiative ‐Depression study uses both the GDS‐SF and the 17‐item Hamilton Depression Rating Scale, a clinician‐administered assessment that rates the presence and severity of symptoms.^46^

### 4.4 Strengths, limitations, and future directions

We expand upon previous studies of the GDS‐SF using a larger and more diverse sample and more statistically robust method for evaluating DIF. This allowed us to probe the influence of a greater number of demographic characteristics, identify both reliable and potentially problematic items, and offer both group‐level and within group comparisons through post‐hoc analyses. The NACC UDS presents a unique opportunity to perform these analyses; however, results must be considered relative to study limitations. First, the NACC sample is best considered a convenience sample of those receiving specialist memory services, and the sample is not representative of the general U.S. population or the population of those with cognitive impairment.^47^ NACC participants are older, better educated, have fewer chronic conditions (i.e., hypertension, diabetes), and endorse fewer depressive symptoms compared to nationally representative samples such as adults aged 60+ from the Health and Retirement Study.^48^ Second, we limited the number of comparison groups within demographic categories by collapsing variables within race and primary language. Given small available sample sizes of select groups, we created Other Race and Other Primary Language groups that include heterogeneous individuals with diverse and varied sociocultural experiences. These combined groups remained smaller in size and resulted in larger standard errors within post hoc analyses, reducing measurement precision. Post hoc results specific to these combined groups may not be generalizable to the communities represented within them,^49^, ^50^ and particularly those communities that represent significant linguistic and cultural diversity.^51^ Ultimately, demographic characteristics, like those utilized within our analyses are proxy measures for intricate and difficult‐to‐measure intersectional sociocultural constructs,^52^ including self‐identified heritage and ancestry, lived experiences, and structural and systemic factors. Version 4 of the NACC UDS strives to address some of these concerns by including additional options for identity characteristics, adding a social determinants of health questionnaire, and providing full translated versions of the UDS battery in both Spanish and Chinese.^53^ Third, our analyses only consider a single, baseline assessment of depression; future work should consider evaluating DIF over time to better understand the risks of measurement bias within longitudinal research. Previous work identifying sources of DIF did not find evidence of widespread “salient DIF” that significantly influenced participant scores on the screening measure, but this work was in smaller samples interrogating fewer characteristics cross‐sectionally.^12^, ^54^ Finally, this paper focuses on the GDS‐SF as a leading screening tool for later‐life depression, without consideration of other mental health conditions and without the ability to clinically verify depression diagnosis. Previous papers utilizing NACC samples have specifically evaluated mental health diagnoses and medication use^55^, ^56^ and noted limitations of the dataset, including the absence of variables regarding diagnostic severity, age of onset, and medication exposure.^57^, ^58^ Future DIF analyses of the GDS may benefit from clinical samples that capture these additional characteristics.

## CONFLICT OF INTEREST STATEMENT

The authors declare no conflicts of interest. Author disclosures are available in the supporting information.

## CONSENT STATEMENT

Participants enrolled at each ADRC provide written consent as part of the IRB‐approved protocol at that site. This consent covers both the data collection procedures required by the respective center as well as the inclusion of the participant's data in the larger NACC UDS database.

## Supporting information



Supporting Information

Supporting Information
