# Supplementary material for: Differential item functioning of the Geriatric Depression Scale‐short form in the NACC dataset
Source: Alzheimers Dement. 2026 Feb 11;22(2):e71114. doi: 10.1002/alz.71114 (PMC12895082; doi:10.1002/alz.71114)
Supplement: Supplementary file 1 — Supporting Information [file ALZ-22-e71114-s002.docx]

**Supplementary Material**

**Supplementary Table 1.** Additional Sample Characteristics

| **Variable** | **Category** | **GDS-SF** | **MoCA** | **MMSE** | **Antidepressant Use** | | **Total Sample**  **N (%)** |
| --- | --- | --- | --- | --- | --- | --- | --- |
|  |  | **Mean (SD)** | **Mean (SD) [N]** | **Mean (SD) [N]** | **No – N(%)** | **Yes – N(%)** |  |
| **Race** | **White** | 2.1 (2.5) | 22.7 (5.7) [10762] | 26.3 (4.2) [10762] | 7895 (73.8) | 2797 (26.2) | 10762 (76.5) |
|  | **Black or African American** | 1.8 (2.2) | 22.4 (4.7) [1867] | 25.6 (4.3) [1867] | 1634 (87.9) | 224 (12.1) | 1867 (13.3) |
|  | **Multiracial** | 2.2 (2.7) | 23.5 (4.5) [390] | 25.6 (4.4) [390] | 315 (80.8) | 75 (19.2) | 390 (2.8) |
|  | **Other** | 2.5 (2.8) | 23.0 (4.5) [444] | 25.3 (4.8) [444] | 385 (87.1) | 57 (12.9) | 444 (3.2) |
| **Ethnicity** | **Not Hispanic** | 2.0 (2.5) | 21.5 (7.4) [12913] | 26.2 (4.4) [12913] | 9838 (76.7) | 2990 (23.3) | 12913 (91.7) |
|  | **Hispanic** | 2.8 (3.0) | 20.2 (7.9) [1106] | 24.5 (5.4) [1106] | 857 (77.8) | 245 (22.2) | 1106 (7.9) |
| **Sex** | **Male** | 2.1 (2.6) | 21.0 (7.2) [6416] | 25.8 (4.4) [6416] | 4976 (77.9) | 1409 (22.1) | 6416 (45.6) |
|  | **Female** | 2.0 (2.5) | 21.7 (7.6) [7661] | 26.3 (4.5) [7661] | 5770 (75.9) | 1833 (24.1) | 7661 (54.4) |
| **CDR** | **No Impairment** | 1.2 (1.9) | 24.1 (7.0) [6053] | 28.7 (2.3) [6053] | 5215 (86.6) | 807 (13.4) | 6053 (43.0) |
|  | **Questionable Impairment** | 2.6 (2.7) | 20.1 (6.3) [5432] | 26.0 (3.5) [5432] | 3907 (72.4) | 1486 (27.6) | 5432 (38.6) |
|  | **Mild Impairment** | 2.9 (2.9) | 13.8 (6.2) [2592] | 20.9 (4.9) [2592] | 1624 (63.1) | 949 (36.9) | 2592 (18.4) |
| **Primary Language** | **English** | 2.0 (2.5) | 21.5 (7.4) [12916] | 26.2 (4.4) [12916] | 9844 (76.7) | 2987 (23.3) | 12916 (91.8) |
|  | **Spanish** | 3.0 (3.1) | 18.9 (7.7) [766] | 24.2 (5.5) [766] | 573 (75.1) | 190 (24.9) | 766 (5.4) |
|  | **Other** | 2.7 (3.0) | 21.8 (6.7) [393] | 25.3 (5.0) [393] | 327 (83.4) | 65 (16.6) | 393 (2.8) |

***Note*.** GDS-SF: Geriatric Depression Scale-Short Form (total possible score range 0-15); MoCA: Montreal Cognitive Assessment (total possible score range 0-30); MMSE: Mini-Mental State Examination (total possible score range 0-30); CDR: Clinical Dementia Rating Dementia Staging Instrument Global score (only participants with scores of 0=no impairment, 0.5=questionable impairment, and 1.0=mild impairment are included in this analysis). Other Race category includes those endorsing Asian/Asian American, Pacific Islander, and American Indian/Alaska Native. Other Primary Language includes those endorsing Mandarin, Cantonese, Russian, Japanese, Other, and Unknown. Not all categories add up to 100% on account of missing demographic data.

**Supplementary Table 2.** Sample Living Situation Characteristics

| **Variable** | **Category** | **Alone** | **Spouse or Partner** | **Relative or Friend** | **Group** | **Other** | **Unknown** |
| --- | --- | --- | --- | --- | --- | --- | --- |
| **Race** | **White** | 2375 (23.2) | 6942 (67.9) | 628 (6.1) | 151 (1.5) | 106 (1.0) | 23 (0.2) |
|  | **Black or African American** | 718 (40.8) | 675 (38.4) | 334 (19) | 20 (1.1) | 9 (0.5) | 2 (0.1) |
|  | **Multiracial** | 112 (30.3) | 181 (48.9) | 54 (14.6) | 16 (4.3) | 6 (1.6) | 1 (0.3) |
|  | **Other** | 77 (19.4) | 254 (64) | 41 (10.3) | 20 (5) | 2 (0.5) | 3 (0.8) |
| **Ethnicity** | **Not Hispanic** | 3140 (25.7) | 7818 (64.1) | 918 (7.5) | 183 (1.5) | 110 (0.9) | 27 (0.2) |
|  | **Hispanic** | 241 (23.8) | 485 (47.9) | 213 (21) | 54 (5.3) | 17 (1.7) | 2 (0.2) |
| **Sex** | **Male** | 761 (12.6) | 4913 (81.5) | 218 (3.6) | 89 (1.5) | 41 (0.7) | 6 (0.1) |
|  | **Female** | 2630 (36.3) | 3432 (47.4) | 915 (12.6) | 148 (2) | 87 (1.2) | 26 (0.4) |
| **CDR** | **No Impairment** | 1834 (32.5) | 3278 (58.1) | 407 (7.2) | 90 (1.6) | 20 (0.4) | 13 (0.2) |
|  | **Questionable Impairment** | 1193 (23.3) | 3402 (66.4) | 388 (7.6) | 76 (1.5) | 51 (1) | 14 (0.3) |
|  | **Mild Impairment** | 364 (14.6) | 1665 (66.6) | 338 (13.5) | 71 (2.8) | 57 (2.3) | 5 (0.2) |
| **Primary Language** | **English** | 3148 (25.8) | 7769 (63.8) | 933 (7.7) | 189 (1.6) | 112 (0.9) | 28 (0.2) |
|  | **Spanish** | 167 (22.9) | 329 (45.2) | 174 (23.9) | 41 (5.6) | 14 (1.9) | 3 (0.4) |
|  | **Other** | 76 (21.2) | 246 (68.7) | 26 (7.3) | 7 (2.0) | 2 (0.6) | 1 (0.3) |

*Note.* Data are shown as N (%).

**Supplementary Table 3.** Sample Level of Independence Characteristics

| **Variable** | **Category** | **Living Independently** | **Some Assistance with Complex Activities** | **Some Assistance with Basic Activities** | **Completely Dependent** | **Unknown** |
| --- | --- | --- | --- | --- | --- | --- |
| **Race** | **White** | 7005 (68.5) | 2430 (23.8) | 682 (6.7) | 50 (0.5) | 58 (0.6) |
|  | **Black or African American** | 1406 (80.0) | 280 (15.9) | 62 (3.5) | 6 (0.3) | 4 (0.2) |
|  | **Multiracial** | 281 (75.9) | 64 (17.3) | 21 (5.7) | 2 (0.5) | 2 (0.5) |
|  | **Other** | 284 (71.5) | 92 (23.2) | 16 (4.0) | 0 (0) | 5 (1.3) |
| **Ethnicity** | **Not Hispanic** | 8589 (70.4) | 2749 (22.5) | 741 (6.1) | 53 (0.4) | 64 (0.5) |
|  | **Hispanic** | 698 (69.0) | 235 (23.2) | 68 (6.7) | 7 (0.7) | 4 (0.4) |
| **Sex** | **Male** | 4000 (66.4) | 1538 (25.5) | 426 (7.1) | 25 (0.4) | 39 (0.6) |
|  | **Female** | 5322 (73.5) | 1458 (20.1) | 385 (5.3) | 36 (0.5) | 37 (0.5) |
| **CDR** | **No Impairment** | 5519 (97.8) | 89 (1.6) | 27 (0.5) | 1 (0.0) | 6 (0.1) |
|  | **Questionable Impairment** | 3359 (65.6) | 1525 (29.8) | 181 (3.5) | 11 (0.2) | 48 (0.9) |
|  | **Mild Impairment** | 444 (17.8) | 1382 (55.3) | 603 (24.1) | 49 (2.0) | 22 (0.9) |
| **Primary Language** | **English** | 8571 (70.4) | 2747 (22.6) | 741 (6.1) | 52 (0.4) | 68 (0.6) |
|  | **Spanish** | 482 (66.2) | 182 (25.0) | 52 (7.1) | 7 (1.0) | 5 (0.7) |
|  | **Other** | 269 (75.1) | 66 (18.4) | 18 (5.0) | 2 (0.6) | 3 (0.8) |

*Note.* Data are shown as N (%).

**Supplementary Table 4.** Sample Type of Residence Characteristics

| **Variable** | **Category** | **Single Family Residence** | **Retirement Community** | **Assisted Living/Boarding Home** | **Skilled Nursing/Nursing Home** | **Unknown** |
| --- | --- | --- | --- | --- | --- | --- |
| **Race** | **White** | 9205 (90.0) | 667 (6.5) | 129 (1.3) | 24 (0.2) | 200 (2.0) |
|  | **Black or African American** | 1631 (92.8) | 78 (4.4) | 11 (0.6) | 1 (0.1) | 37 (2.1) |
|  | **Multiracial** | 345 (93.2) | 16 (4.3) | 3 (0.8) | 1 (0.3) | 5 (1.4) |
|  | **Other** | 372 (93.7) | 11 (2.8) | 7 (1.8) | 0 (0) | 7 (1.8) |
| **Ethnicity** | **Not Hispanic** | 11059 (90.7) | 742 (6.1) | 146 (1.2) | 24 (0.2) | 225 (1.8) |
|  | **Hispanic** | 937 (92.6) | 37 (3.7) | 6 (0.6) | 6 (0.6) | 26 (2.6) |
| **Sex** | **Male** | 5632 (93.4) | 253 (4.2) | 44 (0.7) | 11 (0.2) | 88 (1.5) |
|  | **Female** | 6416 (88.6) | 527 (7.3) | 109 (1.5) | 19 (0.3) | 167 (2.3) |
| **CDR** | **No Impairment** | 5118 (90.7) | 403 (7.1) | 13 (0.2) | 2 (0.0) | 106 (1.9) |
|  | **Questionable Impairment** | 4691 (91.5) | 274 (5.3) | 50 (1.0) | 5 (0.1) | 104 (2.0) |
|  | **Mild Impairment** | 2239 (89.6) | 103 (4.1) | 90 (3.6) | 23 (0.9) | 45 (1.8) |
| **Primary Language** | **English** | 11038 (90.6) | 745 (6.1) | 146 (1.2) | 25 (0.2) | 225 (1.8) |
|  | **Spanish** | 673 (92.4) | 25 (3.4) | 3 (0.4) | 4 (0.5) | 23 (3.2) |
|  | **Other** | 336 (93.9) | 10 (2.8) | 4 (1.1) | 1 (0.3) | 7 (2.0) |

*Note.* Data are shown as N (%).

**Supplementary Table 5.** Sample Marital Status Characteristics

| **Variable** | **Category** | **Married** | **Widowed** | **Divorced** | **Separated** | **Never Married** | **Living as Married** |
| --- | --- | --- | --- | --- | --- | --- | --- |
| **Race** | **White** | 6965 (68.5) | 1686 (16.6) | 908 (8.9) | 62 (0.6) | 396 (3.9) | 154 (1.5) |
|  | **Black or African American** | 689 (39.7) | 515 (29.7) | 352 (20.3) | 43 (2.5) | 124 (7.2) | 11 (0.6) |
|  | **Multiracial** | 191 (52.0) | 80 (21.8) | 60 (16.3) | 8 (2.2) | 26 (7.1) | 2 (0.5) |
|  | **Other** | 266 (68.0) | 65 (16.6) | 40 (10.2) | 2 (0.5) | 14 (3.6) | 4 (1.0) |
| **Ethnicity** | **Not Hispanic** | 7854 (64.8) | 2203 (18.2) | 1271 (10.5) | 95 (0.8) | 529 (4.4) | 162 (1.3) |
|  | **Hispanic** | 529 (52.8) | 207 (20.7) | 149 (14.9) | 40 (4.0) | 66 (6.6) | 11 (1.1) |
| **Sex** | **Male** | 4960 (82.6) | 404 (6.7) | 315 (5.2) | 51 (0.8) | 182 (3.0) | 96 (1.6) |
|  | **Female** | 3462 (48.3) | 2013 (28.1) | 1110 (15.5) | 84 (1.2) | 414 (5.8) | 80 (1.1) |
| **CDR** | **No Impairment** | 3297 (58.9) | 1119 (20.0) | 704 (12.6) | 62 (1.1) | 324 (5.8) | 91 (1.6) |
|  | **Questionable Impairment** | 3427 (67.4) | 800 (15.7) | 539 (10.6) | 47 (0.9) | 206 (4.1) | 64 (1.3) |
|  | **Mild Impairment** | 1698 (68.2) | 498 (20.0) | 182 (7.3) | 26 (1.0) | 66 (2.6) | 21 (0.8) |
| **Primary Language** | **English** | 7801 (64.5) | 2205 (18.2) | 1285 (10.6) | 101 (0.8) | 540 (4.5) | 164 (1.4) |
|  | **Spanish** | 369 (51.3) | 160 (22.3) | 107 (14.9) | 33 (4.6) | 43 (6.0) | 7 (1.0) |
|  | **Other** | 251 (70.7) | 52 (14.6) | 33 (9.3) | 1 (0.3) | 13 (3.7) | 5 (1.4) |

*Note.* Data are shown as N (%).

**Supplementary Table 6.** Item Endorsement by Race

| **Item** | **Endorsement** | **White** | **Black or African American** | **Multiracial** | **Other** |
| --- | --- | --- | --- | --- | --- |
| SATIS | 0 | 9581 (89.03) | 1634 (87.52) | 329 (84.36) | 402 (90.54) |
|  | 1 | 1181 (10.97) | 233 (12.48) | 61 (15.64) | 42 (9.46) |
| DROPACT | 0 | 8654 (80.41) | 1455 (77.93) | 297 (76.15) | 323 (72.75) |
|  | 1 | 2108 (19.59) | 412 (22.07) | 93 (23.85) | 121 (27.25) |
| EMPTY | 0 | 9927 (92.24) | 1709 (91.54) | 340 (87.18) | 390 (87.84) |
|  | 1 | 835 (7.76) | 158 (8.46) | 50 (12.82) | 54 (12.16) |
| BORED | 0 | 9319 (86.59) | 1555 (83.29) | 310 (79.49) | 376 (84.68) |
|  | 1 | 1443 (13.41) | 312 (16.71) | 80 (20.51) | 68 (15.32) |
| SPIRITS | 0 | 10051 (93.39) | 1778 (95.23) | 357 (91.54) | 406 (91.44) |
|  | 1 | 711 (6.61) | 89 (4.77) | 33 (8.46) | 38 (8.56) |
| AFRAID | 0 | 9636 (89.54) | 1729 (92.61) | 350 (89.74) | 353 (79.5) |
|  | 1 | 1126 (10.46) | 138 (7.39) | 40 (10.26) | 91 (20.5) |
| HAPPY | 0 | 9666 (89.82) | 1695 (90.79) | 343 (87.95) | 391 (88.06) |
|  | 1 | 1096 (10.18) | 172 (9.21) | 47 (12.05) | 53 (11.94) |
| HELPLESS | 0 | 9600 (89.2) | 1710 (91.59) | 344 (88.21) | 376 (84.68) |
|  | 1 | 1162 (10.8) | 157 (8.41) | 46 (11.79) | 68 (15.32) |
| STAYHOME | 0 | 8132 (75.56) | 1427 (76.43) | 283 (72.56) | 311 (70.05) |
|  | 1 | 2630 (24.44) | 440 (23.57) | 107 (27.44) | 133 (29.95) |
| MEMPROB | 0 | 7158 (66.51) | 1444 (77.34) | 293 (75.13) | 284 (63.96) |
|  | 1 | 3604 (33.49) | 423 (22.66) | 97 (24.87) | 160 (36.04) |
| WONDRFUL | 0 | 10200 (94.78) | 1831 (98.07) | 378 (96.92) | 417 (93.92) |
|  | 1 | 562 (5.22) | 36 (1.93) | 12 (3.08) | 27 (6.08) |
| WRTHLESS | 0 | 9925 (92.22) | 1753 (93.89) | 364 (93.33) | 397 (89.41) |
|  | 1 | 837 (7.78) | 114 (6.11) | 26 (6.67) | 47 (10.59) |
| ENERGY | 0 | 7420 (68.95) | 1345 (72.04) | 277 (71.03) | 324 (72.97) |
|  | 1 | 3342 (31.05) | 522 (27.96) | 113 (28.97) | 120 (27.03) |
| HOPELESS | 0 | 10198 (94.76) | 1819 (97.43) | 373 (95.64) | 412 (92.79) |
|  | 1 | 564 (5.24) | 48 (2.57) | 17 (4.36) | 32 (7.21) |
| BETTER | 0 | 9954 (92.49) | 1734 (92.88) | 352 (90.26) | 382 (86.04) |
|  | 1 | 808 (7.51) | 133 (7.12) | 38 (9.74) | 62 (13.96) |

*Note.* Data are shown as N (%). Other race group includes those identifying as Asian, American Indian or Alaska Native, and Native Hawaiian or other Pacific Islander.

**Supplementary Table 7.** Item Endorsement by Ethnicity

| **Item** | **Endorsement** | **Not Hispanic** | **Hispanic** |
| --- | --- | --- | --- |
| SATIS | 0 | 11434 (88.55) | 938 (84.81) |
|  | 1 | 1479 (11.45) | 168 (15.19) |
| DROPACT | 0 | 10370 (80.31) | 736 (66.55) |
|  | 1 | 2543 (19.69) | 370 (33.45) |
| EMPTY | 0 | 11900 (92.16) | 897 (81.1) |
|  | 1 | 1013 (7.84) | 209 (18.9) |
| BORED | 0 | 11158 (86.41) | 880 (79.57) |
|  | 1 | 1755 (13.59) | 226 (20.43) |
| SPIRITS | 0 | 12107 (93.76) | 981 (88.7) |
|  | 1 | 806 (6.24) | 125 (11.3) |
| AFRAID | 0 | 11623 (90.01) | 913 (82.55) |
|  | 1 | 1290 (9.99) | 193 (17.45) |
| HAPPY | 0 | 11627 (90.04) | 934 (84.45) |
|  | 1 | 1286 (9.96) | 172 (15.55) |
| HELPLESS | 0 | 11577 (89.65) | 915 (82.73) |
|  | 1 | 1336 (10.35) | 191 (17.27) |
| STAYHOME | 0 | 9830 (76.12) | 729 (65.91) |
|  | 1 | 3083 (23.88) | 377 (34.09) |
| MEMPROB | 0 | 8783 (68.02) | 784 (70.89) |
|  | 1 | 4130 (31.98) | 322 (29.11) |
| WONDRFUL | 0 | 12260 (94.94) | 1062 (96.02) |
|  | 1 | 653 (5.06) | 44 (3.98) |
| WRTHLESS | 0 | 11960 (92.62) | 969 (87.61) |
|  | 1 | 953 (7.38) | 137 (12.39) |
| ENERGY | 0 | 8972 (69.48) | 814 (73.6) |
|  | 1 | 3941 (30.52) | 292 (26.4) |
| HOPELESS | 0 | 12272 (95.04) | 1002 (90.6) |
|  | 1 | 641 (4.96) | 104 (9.4) |
| BETTER | 0 | 11932 (92.4) | 979 (88.52) |
|  | 1 | 981 (7.6) | 127 (11.48) |

*Note.* Data are shown as N (%).

**Supplementary Table 8.** Item Endorsement by Sex

| **Item** | **Endorsement** | **Male** | **Female** |
| --- | --- | --- | --- |
| SATIS | 0 | 5682 (88.56) | 6743 (88.02) |
|  | 1 | 734 (11.44) | 918 (11.98) |
| DROPACT | 0 | 5042 (78.58) | 6108 (79.73) |
|  | 1 | 1374 (21.42) | 1553 (20.27) |
| EMPTY | 0 | 5922 (92.3) | 6931 (90.47) |
|  | 1 | 494 (7.7) | 730 (9.53) |
| BORED | 0 | 5425 (84.55) | 6660 (86.93) |
|  | 1 | 991 (15.45) | 1001 (13.07) |
| SPIRITS | 0 | 5983 (93.25) | 7161 (93.47) |
|  | 1 | 433 (6.75) | 500 (6.53) |
| AFRAID | 0 | 5769 (89.92) | 6821 (89.04) |
|  | 1 | 647 (10.08) | 840 (10.96) |
| HAPPY | 0 | 5783 (90.13) | 6830 (89.15) |
|  | 1 | 633 (9.87) | 831 (10.85) |
| HELPLESS | 0 | 5754 (89.68) | 6790 (88.63) |
|  | 1 | 662 (10.32) | 871 (11.37) |
| STAYHOME | 0 | 4702 (73.29) | 5901 (77.03) |
|  | 1 | 1714 (26.71) | 1760 (22.97) |
| MEMPROB | 0 | 4119 (64.2) | 5489 (71.65) |
|  | 1 | 2297 (35.8) | 2172 (28.35) |
| WONDRFUL | 0 | 6128 (95.51) | 7249 (94.62) |
|  | 1 | 288 (4.49) | 412 (5.38) |
| WRTHLESS | 0 | 5920 (92.27) | 7059 (92.14) |
|  | 1 | 496 (7.73) | 602 (7.86) |
| ENERGY | 0 | 4559 (71.06) | 5261 (68.67) |
|  | 1 | 1857 (28.94) | 2400 (31.33) |
| HOPELESS | 0 | 6058 (94.42) | 7271 (94.91) |
|  | 1 | 358 (5.58) | 390 (5.09) |
| BETTER | 0 | 5851 (91.19) | 7114 (92.86) |
|  | 1 | 565 (8.81) | 547 (7.14) |

*Note.* Data are shown as N (%).

**Supplementary Table 9.** Item Endorsement by Cognitive Status

| **Item** | **Endorsement** | **No Impairment** | **Questionable Impairment** | **Mild Impairment** |
| --- | --- | --- | --- | --- |
| SATIS | 0 | 5595 (92.43) | 4653 (85.66) | 2177 (83.99) |
|  | 1 | 458 (7.57) | 779 (14.34) | 415 (16.01) |
| DROPACT | 0 | 5380 (88.88) | 4067 (74.87) | 1703 (65.7) |
|  | 1 | 673 (11.12) | 1365 (25.13) | 889 (34.3) |
| EMPTY | 0 | 5776 (95.42) | 4858 (89.43) | 2219 (85.61) |
|  | 1 | 277 (4.58) | 574 (10.57) | 373 (14.39) |
| BORED | 0 | 5621 (92.86) | 4479 (82.46) | 1985 (76.58) |
|  | 1 | 432 (7.14) | 953 (17.54) | 607 (23.42) |
| SPIRITS | 0 | 5834 (96.38) | 4941 (90.96) | 2369 (91.4) |
|  | 1 | 219 (3.62) | 491 (9.04) | 223 (8.6) |
| AFRAID | 0 | 5607 (92.63) | 4678 (86.12) | 2305 (88.93) |
|  | 1 | 446 (7.37) | 754 (13.88) | 287 (11.07) |
| HAPPY | 0 | 5635 (93.09) | 4719 (86.87) | 2259 (87.15) |
|  | 1 | 418 (6.91) | 713 (13.13) | 333 (12.85) |
| HELPLESS | 0 | 5751 (95.01) | 4699 (86.51) | 2094 (80.79) |
|  | 1 | 302 (4.99) | 733 (13.49) | 498 (19.21) |
| STAYHOME | 0 | 4843 (80.01) | 3903 (71.85) | 1857 (71.64) |
|  | 1 | 1210 (19.99) | 1529 (28.15) | 735 (28.36) |
| MEMPROB | 0 | 5387 (89) | 2888 (53.17) | 1333 (51.43) |
|  | 1 | 666 (11) | 2544 (46.83) | 1259 (48.57) |
| WONDRFUL | 0 | 5846 (96.58) | 5093 (93.76) | 2438 (94.06) |
|  | 1 | 207 (3.42) | 339 (6.24) | 154 (5.94) |
| WRTHLESS | 0 | 5880 (97.14) | 4883 (89.89) | 2216 (85.49) |
|  | 1 | 173 (2.86) | 549 (10.11) | 376 (14.51) |
| ENERGY | 0 | 4499 (74.33) | 3512 (64.65) | 1809 (69.79) |
|  | 1 | 1554 (25.67) | 1920 (35.35) | 783 (30.21) |
| HOPELESS | 0 | 5912 (97.67) | 5070 (93.34) | 2347 (90.55) |
|  | 1 | 141 (2.33) | 362 (6.66) | 245 (9.45) |
| BETTER | 0 | 5836 (96.42) | 4917 (90.52) | 2212 (85.34) |
|  | 1 | 217 (3.58) | 515 (9.48) | 380 (14.66) |

*Note.* Data are shown as N (%). Cognitive status is taken from the Clinical Dementia Rating (CDR^©^) Dementia Staging Instrument Global score, where 0=No Impairment, .5=Questionable Impairment, and 1=Mild Impairment.

**Supplementary Table 10.** Item endorsement by Primary Language

| **Item** | **Endorsement** | **English** | **Spanish** | **Other** |
| --- | --- | --- | --- | --- |
| SATIS | 0 | 11433 (88.52) | 640 (83.55) | 351 (89.31) |
|  | 1 | 1483 (11.48) | 126 (16.45) | 42 (10.69) |
| DROPACT | 0 | 10363 (80.23) | 491 (64.1) | 295 (75.06) |
|  | 1 | 2553 (19.77) | 275 (35.9) | 98 (24.94) |
| EMPTY | 0 | 11923 (92.31) | 594 (77.55) | 334 (84.99) |
|  | 1 | 993 (7.69) | 172 (22.45) | 59 (15.01) |
| BORED | 0 | 11160 (86.4) | 595 (77.68) | 328 (83.46) |
|  | 1 | 1756 (13.6) | 171 (22.32) | 65 (16.54) |
| SPIRITS | 0 | 12127 (93.89) | 668 (87.21) | 347 (88.3) |
|  | 1 | 789 (6.11) | 98 (12.79) | 46 (11.7) |
| AFRAID | 0 | 11673 (90.38) | 615 (80.29) | 300 (76.34) |
|  | 1 | 1243 (9.62) | 151 (19.71) | 93 (23.66) |
| HAPPY | 0 | 11661 (90.28) | 629 (82.11) | 323 (82.19) |
|  | 1 | 1255 (9.72) | 137 (17.89) | 70 (17.81) |
| HELPLESS | 0 | 11598 (89.8) | 616 (80.42) | 329 (83.72) |
|  | 1 | 1318 (10.2) | 150 (19.58) | 64 (16.28) |
| STAYHOME | 0 | 9822 (76.05) | 496 (64.75) | 283 (72.01) |
|  | 1 | 3094 (23.95) | 270 (35.25) | 110 (27.99) |
| MEMPROB | 0 | 8790 (68.06) | 554 (72.32) | 262 (66.67) |
|  | 1 | 4126 (31.94) | 212 (27.68) | 131 (33.33) |
| WONDRFUL | 0 | 12275 (95.04) | 738 (96.34) | 363 (92.37) |
|  | 1 | 641 (4.96) | 28 (3.66) | 30 (7.63) |
| WRTHLESS | 0 | 11986 (92.8) | 646 (84.33) | 345 (87.79) |
|  | 1 | 930 (7.2) | 120 (15.67) | 48 (12.21) |
| ENERGY | 0 | 8956 (69.34) | 578 (75.46) | 285 (72.52) |
|  | 1 | 3960 (30.66) | 188 (24.54) | 108 (27.48) |
| HOPELESS | 0 | 12285 (95.11) | 681 (88.9) | 362 (92.11) |
|  | 1 | 631 (4.89) | 85 (11.1) | 31 (7.89) |
| BETTER | 0 | 11966 (92.64) | 659 (86.03) | 338 (86.01) |
|  | 1 | 950 (7.36) | 107 (13.97) | 55 (13.99) |

*Note.* Data are shown as N (%). Other primary language includes those endorsing Mandarin, Cantonese, Russian, Japanese, Other, and Unknown.
